# 贝伐珠单抗在晚期非小细胞肺癌一线治疗中的研究进展

**DOI:** 10.3779/j.issn.1009-3419.2020.101.34

**Published:** 2020-07-20

**Authors:** 斌 艾, 轶璠 杨

**Affiliations:** 100730 北京，北京医院，国家老年医学中心，中国医学科学院老年医学研究院，肿瘤内科 Department of Medical Oncology, Beijing Hospital, National Center of Gerontology; Institute of Geriatric Medicine, Chinese Academy of Medical Sciences, Beijing 100730, China

**Keywords:** 贝伐珠单抗, 肺肿瘤, 一线治疗, Bevacizumab, Lung neoplasms, Front line treatment

## Abstract

贝伐珠单抗作为首个抗血管新生的单克隆抗体已经成为晚期非鳞非小细胞肺癌（non-small cell lung cancer, NSCLC）一线治疗的重要组成部分，与化疗、分子靶向治疗以及免疫检查点抑制剂联合使用增强了这些药物的抗肿瘤作用，本文对已有的随机对照临床研究进行综述，希望能为临床治疗工作提供参考。贝伐珠单抗的加入可以显著降低晚期非鳞NSCLC患者的疾病进展风险，特定组合还可以降低患者的死亡风险，最佳组合方案还有待于新的随机对照研究证实。

## Abstract (en)

As the first anti-angiogenesis monoclonal antibody, bevacizumab has been an vital component of front line regimen of advanced nonsqaumous non-small cell lung cancer (NSCLC). It increase the efficacy of chemotherapy, epithelial growth factor receptor tyrosine kinase inhibitors and immune checkpoint inhibitors. This article is an important review of existing randomized controlled clinical research, hoping to provide a reference for clinical treatment. The addition of bevacizumab reduces the risk of disease progression in patients with advanced non-squamous NSCLC significantly. Specific combinations can also reduce the risk of death. The best combination plan needs to be confirmed by new randomized controlled studies.

肿瘤血管新生在癌症的发展、侵袭和转移过程中发挥重要作用，血管内皮生长因子（vascular endothelial growth factor, VEGF）是最重要的促进肿瘤血管新生因子之一。抗血管生成疗法主要是通过使癌症中异常的脉管系统正常化，改善化学药物和其他治疗肿瘤制剂从血液循环到肿瘤组织的运输，以达到增加肿瘤细胞内药物浓度、增强抗肿瘤作用的目的。贝伐珠单抗是最早用于临床的抗血管新生单克隆抗体，它与VEGF结合后阻止了由后者引发的肿瘤血管新生，使异常新生血管正常化从而起到增强其他抗肿瘤药物疗效的作用^[1, 2]^。由于贝伐珠单抗在治疗肺鳞癌时显著增加了出血事件的发生^[3]^，因此，它只可用于非鳞非小细胞肺癌（non-small cell lung cancer, NSCLC）。鉴于贝伐珠单抗在晚期NSCLC一线治疗中发挥了重要的作用，现对其临床研究综述如下。

## 1 贝伐珠单抗与含铂双药化疗的联合

ECOG4599研究^[4]^是首个将贝伐珠单抗纳入晚期NSCLC一线治疗方案的Ⅲ期随机对照研究，化疗组每3周给予200 mg/m^2^的紫杉醇和曲线下面积6剂量的卡铂共6个周期，贝伐珠单抗组每3周给予相同的化疗药物和15 mg/kg的贝伐珠单抗以及贝伐珠单抗维持治疗。贝伐珠单抗组和化疗组的中位生存期（overall survival, OS）、中位无进展生存期（progression free survival, PFS）和客观缓解率（objective response rate, ORR）分别为12.3个月和10.3个月（HR=0.79, *P*=0.003）、6.2个月和4.5个月（HR=0.66, *P* < 0.001）、35%和15%（*P* < 0.001），各项指标的改善均有显著差异，这也是首次将晚期NSCLC的中位OS延长至12个月以上的临床研究。贝伐珠单抗组患者的高血压、蛋白尿、出血、粒细胞缺乏、粒细胞缺乏性发热、血小板减少、低钠血症、皮疹和头痛的发生率显著高于化疗组（*P* < 0.05）。这项研究奠定了贝伐珠单抗在晚期非鳞NSCLC一线化疗中的重要地位。

在中国开展的BEYOND研究^[5]^几乎完全复制了ECOG4599的治疗方案，不同的是紫杉醇的剂量减低为175 mg/m^2^，首要终点也由OS转为PFS，并且采用了随机双盲的试验设计。结果显示加用贝伐珠单抗后显著改善了中位PFS（分别为9.2个月和6.5个月，HR=0.48，*P* < 0.001），ORR（54%和26%，*P* < 0.001）和中位OS（分别为24.3个月和17.7个月，HR=0.68，*P*=0.015, 4）的改善也有统计学差异。表皮生长因子受体（epithelial growth factor receptor, EGFR）突变阳性患者的PFS分别为12.4个月和7.9个月（HR=0.27），中位OS分别为24.3个月和27.5个月（HR=0.90）；野生型患者的中位PFS分别为8.3个月和5.6个月（HR=0.33），中位OS分别为20.3个月和13.8个月（HR=0.57）。基于此项研究我国批准了贝伐珠单抗在晚期非鳞NSCLC的适应证。

与以上两项研究不同，AVAiL研究^[6, 7]^则以顺铂联合吉西他滨作为对照组的化疗方案，并且试验组分别使用7.5 mg/kg和15 mg/kg两种贝伐珠单抗剂量的方案，结果显示无论是低剂量组（6.7个月，HR=0.75，*P*=0.003）还是高剂量组（6.1个月，HR=0.82，*P*=0.03）均较化疗组（6.1个月）的中位PFS显著延长，ORR也显著提高（分别为37.8%，*P*=0.000, 3；34.5%，*P*=0.046和21.6%），但是OS没有显著延长。

鉴于以上研究结果，贝伐珠单抗联合紫杉醇和卡铂的方案，成为治疗晚期非鳞NSCLC的标准一线治疗方案。

由于培美曲塞联合铂类药物及培美曲塞维持治疗在晚期非鳞NSCLC取得总OS获益，因此，两项研究以培美曲塞和卡铂为主体的方案与卡铂联合紫杉醇和贝伐珠单抗的方案进行了比较。对照组均采用了ECOG4599研究中贝伐珠单抗组的治疗方案。在PointBreak研究^[8]^中，研究组使用卡铂联合培美曲塞及贝伐珠单抗15 mg/kg体重并以贝伐珠单抗联合培美曲塞维持治疗，尽管研究组的中位PFS优于对照组（分别为6.0个月和5.6个月，HR=0.83，*P*=0.012），但是没有达到预期的首要终点，即研究组没有获得总OS优势（中位OS分别为12.6个月和13.4个月，HR=1.0，*P*=0.949）。PRONOUNCE研究^[9]^则采用卡铂联合培美曲塞并培美曲塞维持作为研究组的治疗方案，结果显示与对照组比较，无论是是中位OS（10.5个月和11.7个月，HR=1.07）、中位PFS（4.44个月和5.49个月，HR=1.06）还是ORR（23.6%和27.4%，*P*=0.414）均没有优势。

AVAPERL和COMPASS研究则主要比较了维持治疗阶段贝伐珠单抗单药维持与贝伐珠单抗联合培美曲塞维持的疗效。AVAPERL研究^[10, 11]^的诱导化疗阶段使用顺铂联合培美曲塞及贝伐珠单抗7.5 mg/kg的方案，4个周期治疗后疾病未进展的患者随机接受贝伐珠单抗7.5 mg/kg或贝伐珠单抗联合培美曲塞维持治疗，研究的首要终点是自随机开始计算的PFS。结果显示联合维持治疗较贝伐珠单抗单药维持显著延长了患者的PFS，分别为7.4个月和3.7个月（HR=0.48, *P* < 0.001），联合维持治疗的OS延长了3.9个月（分别为17.1个月和13.2个月），但没有统计学差异（HR=0.87, *P*=0.29）。接受联合维持治疗的患者出现了更多的3级以上的不良反应（分别为37.6%和21.7%），其中贝伐珠单抗单药维持者常见为高血压和呼吸困难，联合维持则为粒细胞缺乏、高血压和贫血，且3级以上的血液学毒性仅见于联合治疗组。在日本开展的COMPASS研究^[12]^，诱导方案中化疗药物使用了卡铂和培美曲塞，贝伐珠单抗的剂量为15 mg/kg，4个周期治疗后疾病未进展的患者随机接受贝伐珠单抗或联合培美曲塞的维持治疗，研究的首要终点是自随机开始的OS。虽然联合维持治疗组较单药组的中位OS延长了3.7个月（分别为23.3个月和19.6个月），但是没有达到统计学差异（HR=0.87, *P*=0.069），中位PFS显著延长（分别为5.7个月和4.0个月，HR=0.67，*P* < 0.001），同时联合治疗组的患者也经受了更多严重的不良反应。

ACRIN 5508研究^[13]^则使用传统的卡铂联合紫杉醇及贝伐珠单抗作为诱导治疗方案，4个周期后疾病未进展的患者随机接受贝伐珠单抗、培美曲塞或两者联合的维持治疗，诱导及维持治疗阶段贝伐珠单抗的剂量均为15 mg/kg，首要终点为自随机开始的OS。结果为三组的中位OS分别为14.4个月、15.9个月和16.4个月，单药维持之间以及单药与联合维持相比均没有统计学差异；中位PFS分别为4.2个月、5.1个月（HR=0.85, *P*=0.06）和7.5个月（HR=0.67, *P* < 0.001）；三组3级/4级不良反应发生率分别为37%、29%和51%。研究者认为培美曲塞和贝伐珠单抗维持疗效相同；联合维持治疗由于没有生存获益且毒性显著，所以不宜推荐培美曲塞联合贝伐珠单抗作为维持治疗的方案。

基于上述研究（表 1），我们可以确定与含铂双药方案相比较，增加贝伐珠单抗并作为维持治疗药物，可以显著改善晚期非鳞NSCLC患者的PFS；如果含铂双药方案中使用的是三代化疗药物紫杉醇，那么这种治疗策略也改善了这些患者的OS。在维持治疗阶段，培美曲塞和贝伐珠单抗没有明显的差异；两者的联合与单药维持相比可以延长PFS，但是没有OS获益，并且会发生更多严重的不良反应。鉴于上述研究中，只有AVAiL和AVAPERL两项研究中使用了7.5 mg/kg的贝伐珠单抗，其他研究均使用15 mg/kg的剂量，并且尚无两种剂量对比的随机对照研究，所以临床上仍推荐贝伐珠单抗使用15 mg/kg的剂量。

**1:** 一线应用贝伐珠单抗与含铂双药联合化疗的Ⅲ期随机对照研究 Phase 3 randomized studies of bevacizumab and platinum based double agents chemotherapy as front line treatment for advanced NSCLC

Study	Regimen	*n*	ORR (%)	PFS (mon)	OS (mon)
ECOG 4599	Carboplatin+paclitaxel+bevacizumab 15 mg/kg	434	35.0	6.2	12.3
	Carboplatin+paclitaxel	444	15.0, *P* < 0.001	4.5, HR=0.66	10.3, HR=0.79
AVAiL	Cisplatin+gemcitabine+bevacizumab 15 mg/kg	351	34.6, *P*=0.046	6.5, HR=0.82	13.4, HR=1.03
	Cisplatin+gemcitabine+bevacizumab 7.5 mg/kg	345	37.8, *P*=0.000, 3	6.7, HR=0.75	13.6, HR=0.93
	Cisplatin+gemcitabine	347	21.6	6.1	13.1
PointBreak	Carboplatin+pemetrexed+bevacizumab 15 mg/kg bevacizumab+pemetrexed maintenance	472	34.1	6.0, HR=0.83	12.6, HR=0.949
	Ccarboplatin+paclitaxel+bevacizumab 15 mg/kg bevacizumabmaintenance	467	33.0	5.6	13.4
AVAPERL	Cisplatin+pemetrexed+bevacizumab 7.5 mg/kg then randomization	376	22.7		
	Bevacizumab 7.5 mg/kg+pemetrexed maintenance	128		7.4, HR=0.48	17.1, HR=0.87
	Bevacizumab 7.5 mg/kg maintenance	125		3.7	13.2
BEYOND	Carboplatin+paclitaxel+bevacizumab 15 mg/kg	138	54.0, *P* < 0.001	9.2, HR=0.40	24.3, HR=0.68
	Carboplatin+paclitaxel+placbo	138	26	6.5	17.7
PRONOUNCE	Carboplatin+pemetrexed pemetrexed maintenance	182	23.6, *P*=0.414	4.44, HR=1.06	10.5, HR=1.07
	Carboplatin+paclitaxel+bevacizumab 15 mg/kg bevacizumab maintenance	179	27.4	5.49	11.7
ACRIN 5508	Carboplatin+paclitaxel+bevacizumab 15 mg/kg then randomization	1, 516	30.3		
	Bevacizumab maintenance	287	12.5	4.2	14.4
	Pemetrexed maintenance	294	18.7	5.1, HR=0.85	15.9, HR=0.86
	Bevacizumab+pemetrexed maintenance	293	21.2	7.5, HR=0.82	16.4, HR=0.9
COMPASS	Carboplatin+pemetrexed+bevacizumab 15 mg/kg then randomization	856			
	Bevacizumab maintenance	295		4.0	19.6
	Bevacizumab+pemetrexed maintenance	299		5.7, HR=0.67	23.3, HR=0.87
ORR: objective response rate; PFS: progression free survival; OS: overall survival; NSCLC: non-small cell lung cancer.					

## 2 贝伐珠单抗与EGFR酪氨酸激酶抑制剂的联合

临床前研究表明，与高剂量相比，低剂量的EGFR酪氨酸激酶抑制剂（tyrosine kinase inhibitor, TKI）使得肿瘤细胞发生耐药的时间提前，那么贝伐珠单抗可以使得更多药物进入肿瘤组织中可能会改变这种不利状态^[2, 14]^；EGFR和VEGF有共同的下游信号传导通路，因此同时阻断EGFR和VEGF可能会有更大的临床获益^[15]^；除了有协同抑制作用外，对VEGF的抑制还可以在已经产生*EGFR*耐药突变的肿瘤细胞发挥作用，如克服由T790M导致的耐药^[16]^。基于这些机制，开展了贝伐珠单抗联合EGFR-TKI的临床研究。

JO25567是日本开展的一项开放的Ⅱ期多中心随机对照研究^[17]^，*EGFR*敏感突变阳性的晚期NSCLC患者随机接受口服厄洛替尼联合每3周一次静脉输注贝伐珠单抗或单纯口服厄洛替尼的治疗，联合治疗组的中位PFS较厄洛替尼组显著延长（16.0个月*vs* 9.7个月，HR=0.54，*P*=0.001, 5），但是也出现了更多3度及以上的皮疹（25%和19%）、高血压（60%和10%）和蛋白尿（8%和0%），两组严重不良反应的发生率相似（24%和25%）；两组的ORR（69%和64%，*P*=0.495）和中位OS（47.0个月和47.4个月，HR=0.81）没有统计学差异。在此基础开展的Ⅲ期随机对照研究NEJ026采用了相同的入组条件和相似的治疗方案^[18]^，不同之处在于联合组患者进展后接受卡铂和培美曲塞化疗、培美曲塞维持的治疗方案，厄洛替尼组患者进展后接受卡铂和培美曲塞联合贝伐珠单抗、培美曲塞联合贝伐珠单抗维持的治疗方案。中期分析显示两组的中位PFS分别为16.9个月和13.3个月（HR=0.605）验证了JO25567的研究结果，总生存的数据有待进一步随访。

我国开展的Ⅲ期随机对照研究^[19]^同样纳入*EGFR*敏感突变阳性的晚期NSCLC患者，采用与JO25567相同的治疗方案，联合治疗组较厄洛替尼组的中位PFS显著延长，分别为18.0个月和11.3个月（HR=0.55, *P* < 0.001），缓解持续时间分别为16.6个月和11.1个月（HR=0.57），两组的ORR没有差异（分别为86.3%和84.7%，*P*=0.741）。

所以，已有的研究结果显示，*EGFR*敏感突变阳性的晚期NSCLC患者在一线应用EGFR-TKI的基础上联合贝伐珠单抗，可以显著改善PFS，是否能够改善OS有待于证实。

## 3 贝伐珠单抗与免疫检查点抑制剂的联合

恶性肿瘤的异常血管新生造成了低氧、酸性和高静水压的肿瘤微环境，不但使得化疗药物和免疫检查点抑制剂等抗肿瘤制剂不易到达肿瘤内部发挥作用，同时还利于免疫抑制性细胞的生长和抑制免疫效应细胞的功能，这种环境下某些细胞产生的细胞因子不但在局部促进形成免疫抑制，还释放到循环中促进全身免疫抑制状态的形成。抗血管新生治疗导致的血管正常化可以逆转这种不利局面，不但增加抗肿瘤制剂进入肿瘤内部、改善乏氧状态增加肿瘤对药物和放疗的敏感性，还可以阻断免疫抑制性调节细胞功能，增强免疫效应细胞的功能，从而增强化疗和免疫检查点抑制剂的抗肿瘤作用^[20, 21]^。

IMpower150研究^[22]^是一项开放性Ⅲ期随机对照临床试验，转移性非鳞NSCLC患者随机后分别接受阿托珠单抗加卡铂紫杉醇（ACP组）、贝伐珠单抗加卡铂紫杉醇（BCP组）或在BCP组基础上加阿托珠单抗（ABCP组）治疗4个-6个周期后给予阿托珠单抗、贝伐珠单抗或两者联合的维持治疗。研究结果达到了预设的首要终点，即在野生型意向人群，ABCP组的中位PFS较BCP组显著延长（分别为8.3个月和6.8个月，HR=0.62，*P* < 0.001），其中效应T细胞基因标签高表达的人群也显示ABCP组较BCP组的疾病进展风险显著下降（中位PFS分别为11.3个月和6.8个月，HR=0.51，*P* < 0.001）。即使那些*EGFR*和*ALK*阳性的患者，ABCP组也较BCP组的PFS明显延长（分别为9.7个月和6.1个月，HR=0.59），所有意向人群同样显示ABCP组较BCP组疾病进展的风险明显下降（中位PFS分别为8.3个月和6.8个月，HR=0.72）。总生存的中期分析结果显示野生型意向人群在ABCP组的中位OS较BCP组延长了4.5个月（分别为19.2个月和14.7个月，HR=0.78，*P*=0.02）。ABCP组和BCP组的ORR和中位缓解时间分别为63.5%和48.0%、9.0个月和5.7个月，效应T细胞基因标签高表达者的中位缓解时间分别为11.2个月和5.7个月。两组患者所有不良反应的发生率没有差别，ABCP组有更多的皮疹、口腔炎、粒细胞缺乏性发热和咯血，77.4%发生了免疫相关的不良反应，多数为1级-2级，没有5级毒性出现。

贝伐珠单抗与免疫检查点抑制剂联合在晚期NSCLC一线治疗中的应用有广阔的前景，并且有待于更多临床研究的证实以及发现可以预测疗效的可靠的生物标志。

根据以上临床研究的结果，我们认为贝伐珠单抗在晚期非鳞NSCLC的一线治疗中发挥重要作用，可以显著降低疾病进展的风险，特定情况下的特定组合可以显著改善患者的生存。但是，还有许多问题有待高级别的临床研究进行证实：如不同剂量强度的优劣，缺乏与培美曲塞联合的前瞻性随机对照研究等。除了以上问题外，如何筛选优势人群从而充分发挥其治疗作用、降低治疗相关不良反应的发生率或严重程度，特别是在免疫治疗时代，如何将贝伐珠单抗与其他药物和治疗手段合理组合，制定出适合我国患者的治疗策略，更好地惠及患者，将成为我们重点关注的问题。
